# MESSAGE FRAMING AND ENGAGEMENT IN MENTAL HEALTH CARE: THE MODERATING ROLE OF AGE

**DOI:** 10.1093/geroni/igz038.998

**Published:** 2019-11-08

**Authors:** Shahrzad Mavandadi, Erin Wright, Johanna Klaus, David Oslin

**Affiliations:** Corporal Michael J Crescenz VA Medical Center, Philadelphia, Pennsylvania, United States

## Abstract

Engagement rates in specialty mental health (MH) care for depression are suboptimal across the lifespan, prompting various efforts to promote treatment engagement. This study examined the extent to which age moderates the impact of message framing on appointment attendance among patients referred to specialty MH care. The study employed a randomized, prospective, experimental design. Patients meeting criteria for major depression and referred to specialty MH care at a Veterans Affairs Medical Center (n=360) were randomized to receive either a routine, neutrally worded patient reminder letter prior to their scheduled specialty MH care appointment or a letter that included one of two messages (gain-framed or loss-framed) that were added to the routine letter. Appointment/attendance data were extracted from the computerized patient record system. Logistic regression was used to determine the potential moderating effect of age on the association between message frame and appointment attendance. The sample (mean age = 51.5 (SD=13.5)) was primarily male (85%), non-white (62%), and reported an average depressive symptom score of 19.3 (SD=3.8, range=9-27). Age moderated the impact of message frame on the odds of attending the MH appointment: while younger adults were more likely to attend after receiving a gain-framed message, older adults’ engagement rates did not differ significantly across conditions. Findings suggest that overtly highlighting the benefits of attending an initial specialty MH care appointment, even if in writing, can impact engagement rates among younger adults. Potential alternative, targeted approaches utilizing message framing that may be more effective among older adults should be explored.

